# How I search for a sepsis source

**DOI:** 10.1186/s13054-019-2675-3

**Published:** 2019-11-29

**Authors:** Jan J. De Waele, Yasser Sakr

**Affiliations:** 1 Department of Critical Care Medicine, Ghent University Hospital, C. Heymanslaan 10, 9000 Ghent, Belgium; 2 Department of Anesthesiology and Intensive Care, Uniklinikum Jena, Jena, Germany

**Keywords:** Sepsis, Septic shock, Infection, Diagnosis, Antibiotic therapy

Identifying the infection source in a sepsis patient is important [1] as it allows for better antibiotic choices, recognizes the need for ancillary treatment, and identifies the need for source control interventions [2, 3]. Searching for the source of infection cannot be disconnected from the other aspects of sepsis management [4]. We will start antibiotics based on local guidelines in parallel to the search for the infection source in these patients, but we adopt a watchful waiting strategy in doubtful cases without life-threatening organ failure [5].

We use the following stepwise approach to search for an infection source (Fig. 1).

1. Know your epidemiology

**Fig. 1:**
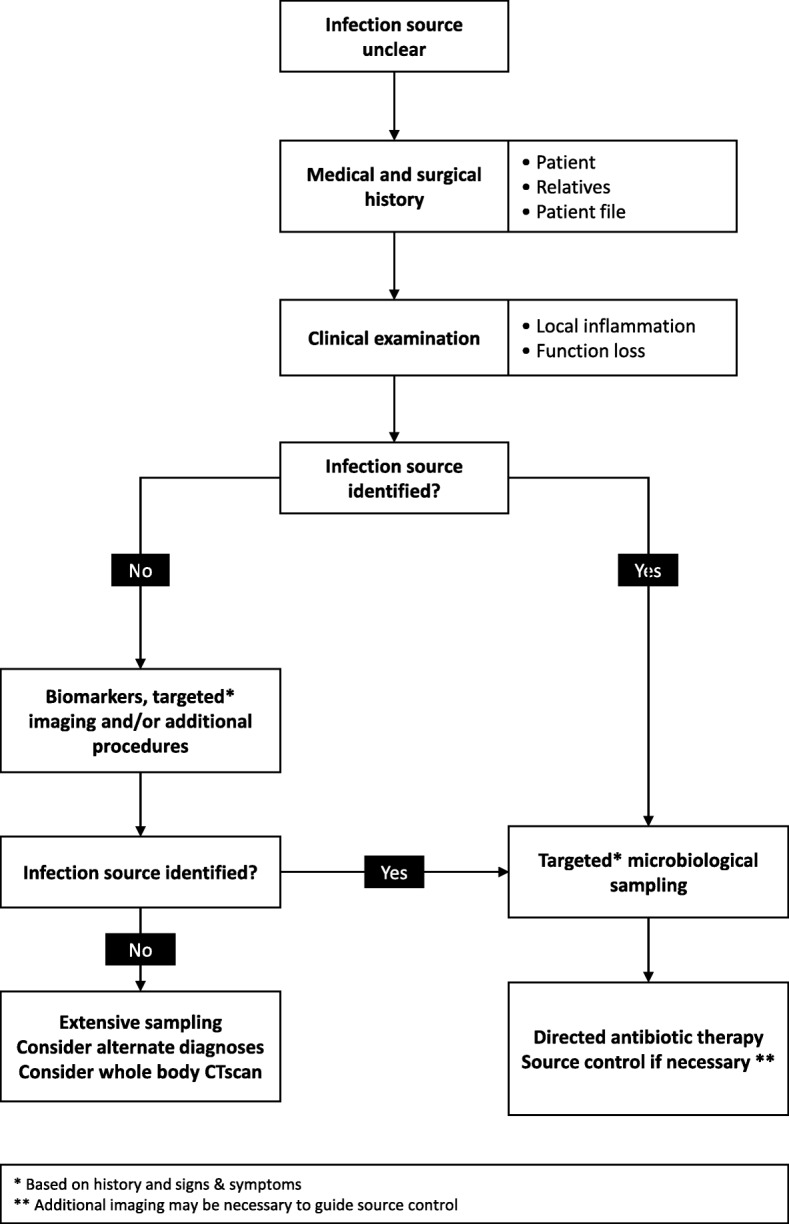
A stepwise approach to identifying the source of sepsis

We integrate the epidemiology of infections in the ICU in the process of searching for infection sources, e.g., respiratory infections are the most common and abdominal infections rank second in most reports [6]. We will also consider the circumstances in which an infection occurs: infections acquired in the ICU are different from infections that are the primary reason for admission.

2. Check the patient’s history

We use the medical and surgical histories to point out the most probable sources of infection or identify other possible diagnoses; this includes presence of risk factors such as substance abuse, diabetes, or recent surgery. We will carefully check for the presence and duration of implantation of foreign devices, e.g., prostheses or intravascular devices.

3. Clinical examination is the key

Local clinical manifestations are more helpful than fever and other systemic signs of inflammation to identify the source of infection. For instance, patients with respiratory infections develop respiratory symptoms such as coughing, increased and purulent sputum, and respiratory insufficiency. In sedated patients or patients with impaired consciousness, we will not rely on symptoms such as pain and clinical examination alone; here, imaging will be more important in the diagnostic process.

We consider meticulous clinical examination, in fact a full head-to-toe inspection, as a critical component of this search. We inspect the body of the patient, auscultate and palpate, and look for painful areas or other signs of local infection. We are not convinced that infection-specific scoring systems (e.g., the Clinical Pulmonary Infection Score) and recognized diagnostic criteria (e.g. modified Duke criteria for infective endocarditis) can be helpful, and we rarely rely on these alone in an ICU patient.

4. Directed imaging

We use imaging selectively and not to confirm obvious infections, unless it is used to plan source control procedures or diagnose the presence of complications, including ultrasound, conventional X-rays, and computed tomography (CT) scan. Ultrasound is primarily helpful for diagnosing specific infections such as cholecystitis or endocarditis; we do not use it alone to diagnose pneumonia or its complications. A conventional chest X-ray is useful to screen for respiratory infections, but not in the diagnostic workup for other sites of infection.

CT scan is the go-to examination for many infections, and we keep a low threshold for (abdominal) CT scans in surgical patients and those with unclear infections. In most patients, IV contrast media will be necessary, possibly complemented with oral or rectal contrast when a GI leak is suspected. Complex cases should be preferably discussed with the attending radiologist to maximize the diagnostic yield of the examination. CT scan may be done if the clinical picture does not improve.

We are not convinced that urgent magnetic resonance imaging (MRI) is helpful unless in severely ill patients with suspected CNS infections that cannot be confirmed on CT scan or using lumbar puncture. Radionucleotide studies may point to undiagnosed systemic dissemination of an infection, but we do not use it in the acute setting.

5. Specific infections require specific approaches

If clinical examination and imaging suggest the presence of more rare and unclear infections, specific diagnostic procedures may be required, e.g., lumbar puncture to diagnose CNS infections.

Similarly, selected high-risk infections may require urgent diagnostic (percutaneous) drainage or surgery to establish the diagnosis. We keep a low threshold for such additional procedures.

6. Laboratory investigations can be helpful

Directed laboratory investigations, with a special emphasis on microbiological sampling and cultures, help to confirm the site of infection. Direct examination of biologic samples may be helpful to detect the presence of microorganisms, inflammatory cells, or biochemical substances.

We use biomarkers such as procalcitonin (PCT) or C-reactive protein (CRP) to aid in diagnosing infection and guiding therapy, but interpret the results with caution, considering the characteristics and kinetics of each biomarker. The relatively rapid decrease in PCT levels of uncomplicated initial insult, such as surgical procedures, can be helpful to point out underlying infections early in this specific context and provoke the early initiation of diagnostic interventions. Therefore, we believe that trends are more important than single observations and that results of any biomarker should always be interpreted in a context of clinical examination and imaging.

7. Timing of investigations and procedures

We seldom see a reason to delay imaging in patients with severe disease or suspected critical infections, such as GI leakage and CNS infections. Particularly when source control is necessary, we will involve a surgeon or (interventional) radiologist upon diagnosis. Depending on the extent of the infection, the complexity of the procedure needed, and the severity of disease, a source control procedure may be performed emergently (Table 1).

**Table 1:** Recommended timing of source control procedures in patients with sepsis and septic shock

Emergent (within 1 h of diagnosis)	Urgent (within 6 h of diagnosis)	Delayed
Necrotizing skin and soft tissue infection debridement	Peritonitis with gastrointestinal leak	Infected pancreatic necrosis
CVC removal	Abdominal abscess	
Wound abscess drainage	Cholecystitis	
Peritonitis with abdominal compartment syndrome	Empyema drainage	

We will base the definitive source identification on the clinical picture, complemented with imaging and/or microbiology confirmation where relevant. This will vary among patients; in some, you will need all three elements, and in a patient with cellulitis or necrotizing infection, the clinical picture may be enough. In conclusion, identifying the source of infection can be challenging, yet important for managing the patient. A stepwise, structured approach helps to do so in minimal amount of time.

## Data Availability

Not applicable

## Abbreviations

ICU: Intensive care unit
CT: Computed tomography
MRI: Magnetic resonance imaging
PCT: Procalcitonin
CRP: C-reactive protein
